# Cologne questionnaire on speechlessness: Development and validation

**DOI:** 10.1007/s12144-022-04102-x

**Published:** 2022-12-11

**Authors:** Thilo Dietz, Sally Tavenrath, Vera Schiewer, Hülya Öztürk-Arenz, Vanessa Durakovic, Hildegard Labouvie, Reinhold S. Jäger, Michael Kusch

**Affiliations:** 1 Department of Internal Medicine I, Faculty of Medicine, Cologne University Hospital, University of Cologne, Kerpener Straße 62, 50937 Cologne, Germany; 2 Centre for Educational Research, University Koblenz-Landau, Campus Landau, Bürgerstraße 23, 76829 Landau in der Pfalz, Germany

**Keywords:** Questionnaire, Emotions, Affective Symptoms, Factor Analysis, Cancer

## Abstract

Speechlessness forms a psychological concept that describes non-speaking or silence in different situations. Speechlessness occurs in particular during emotional stress. The *Cologne Questionnaire on Speechlessness* (ger.: *Kölner Fragebogen zur Sprachlosigkeit*) is an instrument for measuring speechlessness as a function of emotional perception and processing in situations of emotional stress or existing emotional dysregulation. The questionnaire was developed in theoretical proximity to the constructs of alexithymia and expressive suppression. Item selection was performed on a first line sample of *N* = 307 individuals of a normal population. Acquisition of an exploratory model to classify the phenomenon was conducted within four samples in clinical and non-clinical settings. Validation of the factorial structure was performed using an overarching dataset (*N* = 1293) consisting of all samples. The results of a confirmatory factor analysis (CFA) indicated the best model fit (χ^2^ (*df*, 146) = 953.856; *p* < .001; *Tucker-Lewis-Index* = .891; *Comparative Fit Index* = .916; *Root Mean Square Error of Approximation* = .065; *p* < .001; N = 1293) with a four-factorial structure of the questionnaire. Both the overall acceptable validity and reliability recommend the application of KFS on individuals of the normal population as well as clinical subgroups. In addition, the questionnaire can also be used in the context of research on the regulation of emotions.

**Supplementary Information:**

The online version contains supplementary material available at 10.1007/s12144-022-04102-x.

## Introduction

Emotions are fundamental for human behavior. We express our emotions by sharing them with our environment. The primary element of this expression is our speech. Speech forms a complex signal including information about ourselves, our emotions, and requirements to the addressee of the message (Koolagudi et al., 2012). Yet, there are situations where an individual is unable to speak or it remains silent instead of responding. In these cases, that individual appears speechless. Guralnik (1980) defines speechlessness as a state of non-speech or silence of a person that can occur in highly emotional situations or in connection with psychological shock. In a study of 26 psychiatric-hospitalized Holocaust survivors, Laub (2005) recorded a wide range of forms of silence and speechlessness. He described: "They attempted to say something, but it came out as a barely audible scream or a moan." (Laub, 2005, p. 259. The first empirical results on speechlessness can be found in a publication by Berger (2004). Situations that involve distressing emotions are significant for speechlessness. Being speechlessness can therefore be considered an indicator of difficulties in dealing with one's emotions ("For example, speechlessness associated with extreme emotions may be taken as an indicator of individuals' inability to control their emotions […]" Berger, 2004, p. 174). This non-speaking or silence can lead to a cycle of increasing insecurity (Canzona et al., 2019), growing isolation (Atkins et al., 2013; Konradsen et al., 2012) and even mental disorders (Paoletti & Ben-Soussan, 2020).

### Alexithymia, expressive suppression, and speechlessness

The inability to perceive and describe emotions with words has been described for more than 50 years in relation to the various forms of alexithymia (Bagby et al., 2020; Lane et al., 2020; Nemiah & Sifneos, 1970). Individuals with marked expressive suppression also appear to not only suppress their emotions, but show associated limitations in speech production and verbal conversation (Goerlich-Dobre et al., 2014; Peters et al., 2014; Roche & Arnold, 2018) which in turn is associated with the use of fewer words as well as increased silence (Butler et al., 2003; Gross, 2002; Kim, 2008; Richards et al., 2003). Using the Toronto Alexithymia Scale-26 (TAS-26; Kupfer et al., 2001) and the Emotion Regulation Questionnaire (ERQ; Abler & Kessler, 2009), Schiewer et al. (2022) were able to show that a common factor underlies the constructs of alexithymia and expressive suppression. The authors thus confirmed the factorial relationship previously recorded by Kessler et al. (2010), but not defined as an independent construct. Continuing, the authors designate this factor as "speechlessness" (Schiewer et al., 2022, p. 171), as reduced speech behavior (non-speech or silence) is implicated in dysfunctional emotion processing or emotional dysregulation (in the form of alexithymia and/or expressive suppression) (cf. Berger, 2004), whereas the latter manifest as an absence of flexibility in managing and responding to emotions (Werner & Gross, 2010).

### Emotional distress and speechlessness in cancer

Research on emotional dysregulation indicate a strong association to chronic diseases (Ciuluvica et al., 2014, 2019; Wierenga et al., 2017). Cancer is one of the most common and serious chronic diseases. An oncologic diagnosis and events during the disease progression and cancer therapy are often experienced by patients as traumatizing situations (Gieseler et al., 2018; Kusch et al., 2012; Palmer et al., 2004). In their work, Shands et al. (1951) refer to patients reporting "they were rendered speechless for a while." (Shands et al., 1951, p. 1160). Research on verbal expression of emotions shows that cancer patients are significantly more likely to have difficulty putting their feelings into words, ranging from 26% to 42.5% (DeVries et al., 2012), compared to the general population, ranging from 5.2% to 17% (Franz et al., 2008). In addition, the stigma of "cancer" can be associated with silence and feelings of isolation for those affected (Daher, 2012) which is often observed in family communication (Zhang & Siminoff, 2003).

### Aim of the work

Speechlessness manifests as a non-speaking or silence especially in the context of emotional distress (Berger, 2004) and emotional dysregulation (Schiewer et al., 2022). Individuals with a chronic (Ciuluvica et al., 2014, 2019; Wierenga et al., 2017) or oncological disease (DeVries et al., 2012), psychosomatic disorders (Bagby et al., 2020; Lane et al., 2020) or states of emotional dysregulation (Schiewer et al., 2022) show increased difficulties to put their emotions into words.

The *Cologne Speechlessness Questionnaire* (ger.: *Kölner Fragebogen zur Sprachlosigkeit*—KFS) was designed to assess emotional speechlessness in the context of emotionally stressful situations and/or life events. The psychometric survey instrument on speechlessness is intended to describe the different facets of the difficulty in putting emotions into words and to serve for the identification of individuals with a high expression of speechlessness. The questionnaire refers to theoretical concepts of emotional dysregulation and translates them into a non-pathological concept in the phenomenon of speechlessness. The phenomenon of speechlessness captured by the KFS is distinguished from somatic or psychopathological concepts by not quantifying the degree or presence of an individual's pathology, but by enabling a tangible understanding of the extent to which the individual is affected by speechlessness over one's emotions or by speechlessness due to an overwhelming of one's emotions. To the best of the authors' knowledge, no corresponding instrument for recording speechlessness is consisting in approach yet.

The aim of this work is to outline the development of the questionnaire, its items and to validate the instrument in order to make it usable in psychotherapeutic, psychosomatic and psycho-oncological care.

### Methodology

#### Questionnaire development

The questionnaire was developed by a multidisciplinary team consisting of psychologists, psychotherapists and healthcare service scientists. In a first step, 24 items were formulated in semantic proximity to the constructs of alexithymia according to the German version of the TAS-26 (Kupfer et al., 2001) and the Perth Alexithymia Questionnaire (PAQ; Preece et al., 2018) as well as expressive suppression, based on the German version of the ERQ (Abler & Kessler, 2009). For a total of four of the 24 items, the formulation was independent of a semantic proximity to the aforementioned instruments. The focus of these items was on the emotional attention or perception of one's own emotions in different emotional conditions.

#### Measurement instruments

The TAS-26 (Kupfer et al., 2001) forms a validated, German questionnaire for assessing alexithymia with a total of 26 items on a 5-point Likert scale. It includes three subscales (1) Difficulty in Identifying Feelings (DIF), (2) Difficulty in Describing Feelings (DDF), and (3) Externally-Oriented Thinking Style (EOT) and an Alexithymia sotal scale. The subscales of the TAS-26 (Kupfer et al., 2001) replicate the Toronto Model construct (Taylor et al., 1997). The internal consistency of the total and subscales of the TAS-26 (Kupfer et al., 2001) from sample 1 (*N* = 203 complete data) were α = 0.88 (DIF), α = 0.83 (DDF), α = 0.57 (EOT), and α = 0.85 (alexithymia total scale).

The PAQ (Preece et al., 2018) assesses alexithymia using five subscales (Negative-Difficulty identifying feelings, N-DIF; Positive-Difficulty identifying feelings, P-DIF; Negative-Difficulty describing feelings, N-DDF; Positive-Difficulty describing feelings, P-DDF; General-Externally oriented thinking, G-EOT), with a total of 24 items on a 7-point Likert scale according to the Attention-Appraisal Model (Preece et al., 2017). Because the PAQ is currently only available in an English-language version, it was not part of the survey instrumented administered within the samples.

The ERQ (Abler & Kessler, 2009) measures the two emotion regulation strategies (1) expressive suppression (ES) and (2) cognitive reappraisal (CR) using 10 items on a 7-point Likert scale according to the theory of emotion regulation (Gross, 2014). The scale reliability of sample 1 and 2 of the ES scale was α = 0.80 (sample 1 [*N* = 208]) and α = 0.78 (sample 2 [*N* = 383]), respectively, and that of the CR scale was α = 0.84 (sample 1 [*N* = 208]) and α = 0.86 (sample 2 [*N* = 382]).

#### Data

**Fig. 1:**
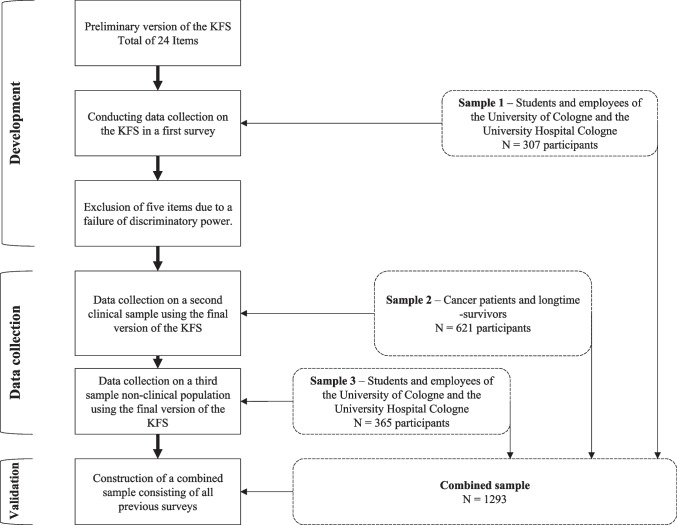
Composition of the data set

Since the development of the KFS in April 2020, the questionnaire has been used in a total of three surveys until March 2022. These surveys form the data set for the analyses reported in this paper (cf. Fig. 1). Two surveys (sample 1 and sample 3) were conducted in a convenience sample of students and employees of the University of Cologne and the University Hospital Cologne. Another survey (sample 2) were conducted among cancer patients and long-term survivors.

Sample 1 includes a total of *N* = 307 participants. The survey period ranged from July to November 2020. Individuals were contacted via university- or hospital-internal mail distribution lists. Participants in sample 1 received the preliminary version of the KFS with a total of 24 items (Fig. 1). A total of 73.6% (*N* = 226) of participants provided complete information on all items of the KFS. Additionally, a total of *N* = 203 (66.1%) participants from sample 1 provided complete information on TAS-26 (Kupfer et al., 2001) and *N* = 208 (67.8%) provided complete information on ERQ (Abler & Kessler, 2009). The mean age of women (83.7%) and men (16.3%) was M = 36.78 years (SD = 12.70; range = 18—68). The majority had a university degree at 55.4% (bachelor's degree: 13.4%; master's degree: 17.8%; diploma: 8.4%; promotion/PhD: 15.8%), vocational training at 13.4%, and a high school diploma at 31.2% (ESM Table E1).

The second survey (sample 2) included a total of *N* = 621 oncological patients in acute therapy as well as long-term survivors. The survey period ranged from March to August 2021. The sample was conducted from members of cancer self-help groups contacted throughout Germany. Participants received the selected version of the KFS consisting of a total of 19 items (cf. Fig. 1). In addition, subjects were administered the ERQ (Abler & Kessler, 2009). A total of 75.5% (*N* = 469) of individuals provided complete information on items of the KFS. Complete information on the ERQ was provided by *N* = 382 (61.5%). The mean age of women (78.1%) and men (21.9%) was M = 55.8 years (SD = 11.57; range = 20—84). Sociodemographic information are obtained in ESM Table E1.

Recruitment in sample 3 was analogous to recruitment in sample 1. This was supplemented by an enquiry to employees of the University Hospital Cologne to participate in the study via the company's internal communication portal. The survey period ranged from October 2021 to March 2022. A total of *N* = 365 individuals took part in the survey using the final version of the KFS (cf. Fig. 1). A total of 63.6% (*N* = 232) individuals provided complete information on items of the KFS. The mean age of women (82.7%) and men (17.3%) was M = 34.39 years (SD = 13.62; range = 18—65). One third (33.9%) of the participants had a university degree (bachelor's degree: 12.6%; master's degree: 9.8%; diploma: 4.6%; doctorate: 12.6%). Vocational training was indicated by 16.4% of the respondents. The majority of respondents (49.7%) indicated high school diploma (35.9%) or lower as their highest educational qualification (ESM Table 1).

To validate the questionnaire, the data from all three surveys were combined into a final sample (cf. Fig. 1). This combined sample consisted of *N* = 1293. Complete information on KFS items was given for 71.7% (*N* = 927). The proportion of female participants was 80.6%. The mean age of all individuals included in the data set was M = 45.28 years (SD = 16.17; range = 18—84). Detailed sociodemographic information are obtained in ESM Table E1.

#### Analysis

Data analysis was performed using IBM SPSS Statistics Version 27 and IBM SPSS Amos Version 27. The data was analyzed stepwise.

In the first step, selection of the preliminary version of the KFS was performed on the basis of item difficulty, discriminant power and scale reliability is exclusively in sample 1 (cf. Fig. 1). Item selection was based on item difficulty, item discriminant power, and scale reliability (Chronbach’s Alpha). Item difficulty (${P}_{i}$) was calculated using the formula ${P}_{i}=\frac{{x}_{i}}{max({x}_{i})}\bullet 100$, where ${x}_{i}$ is the mean item value and ${max(x}_{i})$ is the maximum achievable value in item $i$ (Kelava, 2010). Item discriminant power and Cronbach’s Alpha were calculated using the item analysis options available in SPSS (cf. Kelava, 2010). The calculation of item discriminant power was performed by using the formula ${r}_{i(t-i)}= \frac{\sigma ({x}_{i}, {x}_{t-i})}{\sigma ({x}_{i})\bullet \sigma ({x}_{t-i})}$, where ${x}_{i}$ is the value based on all items $i$ and ${x}_{i}$ is the value in the overall test without item $i$ (cf. Fisseni, 2004). According to standards of literature (Kelava & Moosbrugger, 2020), item difficulty indices between *5* and *95* were chosen to capture extreme trait expressions, as well as a discriminant power of the items within the value range of *0.4—0.7*. Cronbach’s Alpha > *0.7* was targeted for scale reliability (cf. Tavakol & Dennick, 2011). Items with values below or above the pre-set thresholds were excluded. Subsequently, the item analysis was performed again.

The second step involved performing exploratory factor analysis (EFA) on all samples as well as the combined sample. Due to the exploratory nature of speechlessness, the theoretical classification and the pursued approach of a non-pathological concept, EFA was conducted using principal components analysis (PCA), oblique rotation (oblimin, direct), and an eigenvalue of > 1. The aim of this procedure was the extraction of individual factors of speechlessness in the context of emotional extreme situations and emotional dysregulation. EFA was performed for each sample.

The third and final step involved the validation of the questionnaire. In order to obtain the best model to represent the phenomenon of speechlessness, testing of the factor structure extracted using the EFA was performed on all samples as well as the combined sample using confirmatory factor analyses (CFA). The CFA was performed using IBM SPSS Amos 27 Graphics. The background of the procedure was the formation of robust factors on which a concept of speechlessness in the context of emotional perception and processing could be based. The model was estimated using maximum likelihood (ML) methods. Missing data were estimated by using the Full Information Maximum Likelihood (FIML) provided by Amos. The FIML method estimates relevant sample parameters (e.g. covariances, variances). No imputation of missing data at the individual level is performed, which could result in potential bias (Arbuckle, 2019; Jekauc et al., 2012). The Tucker-Lewis coefficient (TLI), Comparative Fit Index (CFI), and Root Mean Square Error of Approximation (RMSEA) were used to test model fit. The analysis and evaluation of the results was based on the specifications of Whittaker (2016), Hu and Bentler (1995): CFI > *0.9* (Whittaker, 2016) and RMSEA < *0.10* (Hu & Bentler, 1995). Gender difference for the subscales of the KFS were analyzed using a two-tailed t-test for independent samples.

Structural equation modeling (SEM) was conducted to examine potential measurement invariance (MI; cf. Mellenbergh, 1989) due to various contextual factors (samples and timing of surveys). The basis for these calculations was the proportion of female participants from all samples due to the predominance of this gender in each sample (cf. ESM Table E1). Distinctions in groups were based on clinical and non-clinical female participants. MI between the participants was evaluated using Cheung and Rensvold (2002) and their recommend deviation of CFI (ΔCFI) ≤ 0.01. The fundamental four steps (1) *configural*, (2) *metric*, (3) *scalar* and (4) *residual*, according to literature (Putnick & Bornstein, 2016), were performed.

In addition, construct validity was tested using Pearson-Product-Moment-Correlation (PPMC). For this purpose, the final extracted scales of the KFS were correlated with the sub- and total scales of the TAS-26 (Kupfer et al., 2001) and the ERQ (Abler & Kessler, 2009). Since a development of the items was semantically close to these two survey instruments, as well as the results around Schiewer et al. (2022) showed a correlative and factorial relationship of both theoretical concepts measured by the constructs of the TAS-26 (Kupfer et al., 2001) and the ERQ (Abler & Kessler, 2009), a high correlation to both instruments was aimed for.

## Results

### First step – item analysis

The item difficulty of all 24 items was within the specified range of 5 to 95. Items 14 to 18 scored negative discriminant power (-0.046 to -0.188). Items 20 to 24 scored a discriminant power of < 0.4. Due to the results, Item discriminant power was calculated in a second pass using recoded items 14 to 18 and excluding items 20 to 24. The items achieved a positive value, which met the specifications for discriminatory power (ESM Table 2). The final version of the KFS contains 19 items. The internal consistency of all selected items was Cronbach's α = 0.923. Item difficulty and discriminatory power are presented in ESM Table 2.

### Second step – exploratory factor analysis

A total of four Exploratory factor analysis (EFA) were performed. For data from sample 1 (*N* = 226) a four-factorial structure could be extracted at a Kaiser–Meyer–Olkin (KMO) criterion of 0.909 (χ^2^ (df, 171) = 2592.54; *p* < 0.001) and a resolved total variance of 68.42% (Tab 1). Within the sample of oncology patients (sample 2, *N* = 469), a three factorial structure was extracted with a KMO of 0.932 (χ^2^ (df, 171) = 4562.57; *p* < 0.001) and a resolved total variance of 60.01% (Table 1). Sample 3 (*N* = 232) achieved a three-factor structure with a KMO of 0.903 (χ^2^ (df, 171) = 2667.131; *p* < 0.001), with a total variance resolved of 62.55% across all factors (Table 1). Using the data from the combined sample (*N* = 927), a three factorial structure was extracted with a total variance resolved of 61.96% with a KMO of 0.937 (χ^2^ (df, 171) = 9722.065; *p* < 0.001; Table 1). The KMO values of all EFAs indicated a good fit of the data (cf. Kaiser & Rice, 1974). The highest total variance resolved was identified in a four-factorial structure in sample 1.

**Table 1:** Exploratory factor analysis

	Sample 1 (*N* = 226)	Sample 2 (*N* = 469)	Sample 3 (*N* = 232)	Combined sample (*N* = 927)
*Kaiser–Meyer–Olkin*	.909	.932	.903	.937
*χ*^*2*^* (df)*	2592.540 (171)	4562.57 (171)	2667.131 (171)	9722.065 (171)
*p*	< .001	< .001	< .001	< .001
*Cumulated variance (in percent)*	68.42%	60.01%	62.55%	61.96%
*Factors*	4	3	3	3

Values < .3 are not shown for better readability. Exploratory factor analysis using the principal components method based on an eigenvalue > 1 and an oblique rotation (oblimin, direct) with delta = 0

### Third step – validation

Confirmatory factor analysis was used to test the best model fit of the extracted factors (cf. ESM Table E3). For this purpose a total of four models were formed according to the order of the EFAs performed (Model 1 = EFA sample 1; Model 2 = EFA sample 2; Model 3 = EFA sample 3; Model 4 = EFA combined sample; cf. Table 1; ESM Table E3). CFA were performed separately for data from the normal population (sample 1 + sample 3) and data from the clinical sample (sample 2), as well as the combined sample (Table 2).

**Table 2:** Confirmatory factor analysis

	Non-cancer individuals (*N* = 672; Sample 1 and 3)				Cancer individuals (*N* = 621; Sample 2)				Combined Sample (*N* = 1293)			
	Model 1	Model 2	Model 3	Model 4	Model 1	Model 2	Model 3	Model 4	Model 1	Model 2	Model 3	Model 4
*χ*^*2*^	731.022	1070.543	836.208	935.807	437.459	583.328	534.778	553.483	953.856	1389.292	1170.507	1278.282
*χ*^*2*^*/df ratio*	5.007	7.09	5.612	6.281	2.996	3.863	3.589	3.715	6.533	9.2	7.856	8.579
*CFI*	.887	.823	.867	.848	.935	.903	.913	.909	.916	.872	.894	.883
*TLI*	.853	.777	.831	.806	.915	.878	.889	.884	.891	.839	.865	.851
*RMSEA*	.077***	.095***	.083***	.089***	.057*	.068***	.065***	.066***	.065***	.080***	.073***	.077***

*CFI* comparative fit index, *TLI* Tucker-Lewis coefficient, *RMSEA* root mean square error of approximation; * *p* < .05; ** *p* < .01; ***p < .001; In Model 3, KFS item I6 was assigned to the first factor and was not separated, considered as a stand-alone factor

Considering Wheaton et al. (1977), model 1 yielded the best result within the sample of non-cancer individuals and the sample of cancer individuals, with χ^2^/df-ratio = 5.007 and χ^2^/df-ratio 2.996, respectively (Table 2). Within the combined sample, model 1 also scored the best compared to the other models, but it scored 6,533, which was higher than the < 5 required by Wheaton et al. (1977). Considering the CFI, TLI and RMSEA, model 1 achieved the results required within the literature (Hu & Bentler, 1995; Whittaker, 2016) and showed the highest model performance compared to the other models (Table 2).

Within the non-cancer individuals sample, the CFI (0.887) and TLI (0.853) scored minimal below the recommended CFI/TLI > 0.9 by Whittaker (2016). The RMSEA scores 0.077 and was below the recommended RMSEA < 0.10 by Hu and Bentler (1995). Model 1 achieved the best CFI (0.935), TLI (0.915), and RMSEA (0.057) values within the cancer individuals sample (Table 2). These were within the range of values required by the literature (Hu & Bentler, 1995; Whittaker, 2016). This also applied to the values (CFI = 0.916; RMSEA = 0.066) of the combined sample with the exception of the TLI, which at 0.891 was slightly below the required TLI > 0.9 (Table 2).

**Fig. 2:**
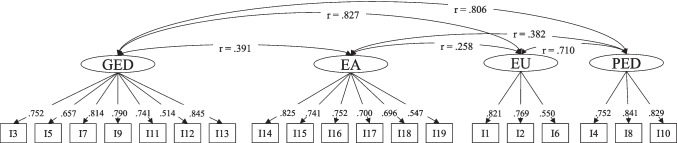
Confirmatory factor analysis with factor loads for the four subscales of the KFS. Legend. *N* = 1293; r = correlations between the factors; The values on the arrows represent the standardized regression weights; GED = General Emotion Description; EA = Emotional Awareness; EU = Emotional Uncertainty; PED = Positive Emotion Description

Based on the results of the CFA (Table 2) and according to the best model fit, model 1 was chosen as the factorial model for the KFS. According to the results of the model pattern matrix of model 1 (ESM Table E3), the 19 items were assigned to the four factors. Each factor was assigned a corresponding subscale with the following names: 1^st^ factor *General Emotion Description* (GED; ger.: *Allgemeine Gefühlsbeschreibung*), factor 2^nd^ factor *Emotional Awareness* (EA; ger.: *Gefühlsbeachtung*), 3^rd^ factor *Emotional Uncertainty* (EU; ger.: *Gefühlsunsicherheit*), and 4^th^ factor *Positive Emotion Description* (PED; ger.: *Positive Gefühlsbeschreibung*). In addition, all items were combined in a total score. A visual representation of the factorial structure of the KFS as well as the standardized regression weights and the correlation of the factors among each other are presented in Fig. 2. Considering the combined sample internal consistency of the subscales were:

1. *General Emotion Description* (GED): Cronbach’s Alpha = 0.890 (*N* = 927; complete information on the KFS obtained from the combined sample)
2. *Emotional Awareness* (EA): Cronbach’s Alpha = 0.853 (*N* = 927; complete information on the KFS obtained from the combined sample)
3. *Emotional Uncertainty* (EU): Cronbach’s Alpha = 0.750 (*N* = 927; complete information on the KFS obtained from the combined sample)
4. *Positive Emotion Description* (PED): Cronbach’s Alpha = 0.850 (*N* = 927; complete information on the KFS obtained from the combined sample)

The KFS total score, consisting of all 19 items achieved a Cronbach’s Alpha = 0.916 (*N* = 927; complete information on the KFS obtained from the combined sample). Specific results on scale reliability for each sample can be found in Table ESM E4.

Due to the high correlations between the factors GED and EU, GED and PED, and EU and PED, an additional CFA was conducted using the combined sample data to test a single factorial model. The single factorial model scored a CFI = 0.7, a TLI = 0.625 and a RMSEA = 0.122 with a χ^2^/df- ratio = 20.09 (χ^2^(152) = 3053.786; *p* < 0.001), indicating a low model fit. To eliminate potential bias in the results based on the unequal gender distribution, additional CFA was conducted based on the final factor structure of the KFS separated for men (*N* = 207) and women (*N* = 861). CFI (0.883) and TLI (0.848) of the male sample were minimally below the requirements of Whittaker (2016). This was also true for the TLI (0.889) of the female sample (ESM Table E5). The RMSEA of both samples (male = 0.066; female = 0.072) met the requirements (ESM Table E5).

Before SEM was conducted to assess MI, CFA was calculated for nonclinical and clinical female participants. For non-cancer female participants (*N* = 455) the model scored CFI = 0.883; TLI = 0.848, and RMSEA = 0.085 with a χ^2^ (146) = 624.395(*p* < 0.001). For cancer female participants (*N* = 406) the model scored CFI = 0.927; TLI = 0.905, and RMSEA = 0.064 with a χ^2^ (146) = 384.615 (*p* < 0.001). The baseline model for configural invariances fulfills the basic requirements for model fit (CFI > 0.9; cf. Hu & Bentler, 1995; ESM Table E7). The test for metric invariances indicated a significant worsening in model fit (Δχ^2^ = 425; *p* > 0.001; ΔCFI = 0.056). According to Cheung and Rensvold (2002) ΔCFI was below ≤ 0.01. Also the tests for scalar and residual invariances indicated significant deteriorations in χ^2^ (*scalar invariance*: Δχ^2^ = 173.3; *p* > 0.001; *residual invariance*: Δχ^2^ = 164.3; *p* > 0.001) and CFI (*scalar invariance*: ΔCFI = 0.021; *residual invariance*: ΔCFI = 0.02; ESM Table E7). The results also indicated partial MI (ESM Table E6). A better model fit was obtained with free estimation of the intercepts for items 2, 4, 5, 6, and 19, while the other intercepts were held constant (ESM Table E6).

### Psychometric characteristics

Non-cancer individuals (sample 1 and 3) scored a mean M = 32.42 (SD = 17.93) on the Speechlessness total score, and cancer individuals (sample 2) scored M = 41.18 (SD = 18.01). Within the combined sample, participants scored a mean M = 36.85 (SD = 18.49; Table 3).

**Table 3:** Psychometric characteristics of non-cancer and cancer individuals and combined sample

	Non-cancer individuals (Sample 1 and 3)				Cancer individuals (Sample 2)				Combined sample			
	N	M	SD	Range	N	M	SD	Range	N	M	SD	Range
*GED*	458	13.37	8.55	0 – 34	469	15.88	8.83	0 – 35	927	14.64	8.78	0 – 35
*EA*		10.79	6.47	0 – 27		13.31	6.68	0 – 30		12.06	6.7	0 – 30
*EU*		4.91	3.71	0 – 15		6.9	3.86	0 – 15		5.92	3.91	0 – 15
*PED*		3.36	3.4	0 – 15		5.09	3.93	0 – 15		4.23	3.77	0 – 15
*Total score*		32.42	17.93	0—86		41.18	18.01	0 – 87		36.85	18.49	0 – 87

*M* mean, *SD* standard deviation, *GED* general emotion description, *EA* emotional awareness, *EU* emotional uncertainty, *PED* positive emotion description

Results of the t-test for gender group differences revealed significant mean differences for cancer individuals between men (*N* = 108) and women (*N* = 361) for the subscales GED (*t*(467) = –3.902; *p* < 0.001), EU (t(467) = -4.170; *p* < 0.001) as well the KFS total Score (*t*(467) = –5.09; *p* = 0.01) indicating higher mean values for female participants. t-test for gender group differences between male (*N* = 69) and female (*N* = 364) non-cancer individuals revealed only for the subscale PED significant differences (*t*(431) = 2.423; *p* = 0.016) indicating higher mean values for male participants.

Zero-order correlations between the subscales of the KFS showed consistently significant, moderate to strong positive correlations for sample 1 (|r|> 0.3) and sample 2 (Table 4). The GED and PED subscales achieved the highest correlations in both samples (sample 1: r = 0.649; *p* < 0.001; sample 2: r = 0.737; *p* < 0.001; Table 4). In sample 1, significant, strong positive correlations (|r|> 0.5) were evident between the KFS total score and the DIF and DDF subscales, as well as the Alexithymia total score of the TAS-26 (Kupfer et al., 2001). In both sample 1 and sample 2, a significant, moderate to strong positive correlation between the (sub)scales of the KFS and the ES scale of the ERQ (Abler & Kessler, 2009) could be identified (Table 4). In addition, significant, weak negative correlations (r = -0.119 to r = -0.278; Table 4) were identified between the KFS subscales and the CR subscale of the ERQ (Abler & Kessler, 2009).

**Table 4:** Pearson product-moment correlations of zero order as well as between the (sub-) scales of the KFS and the scales of the TAS-26 as well as the ERQ

	Sample 1					Sample 2				
	(1) KFS – GED	(2) KFS – EA	(3) KFS – EU	(4) KFS – PED	(5) KFS – Total score	(1) KFS – GED	(2) KFS – EA	(3) KFS – EU	(4) KFS – PED	(5) KFS – Total score
*(1)*	1	–	–	–	–	1	–	–	–	–
*(2)*	.503**	1	–	–	–	.301**	1	–	–	–
*(3)*	.624**	.336**	1	–	–	.682**	.116*	1	–	–
*(4)*	.649**	.425**	.489**	1	–	.737**	.287**	.569**	1	–
*TAS – DIF*^*a*^	.690**	.450**	.710**	.548**	.736**	N.A	N.A	N.A	N.A	N.A
*TAS – DDF*^*a*^	.813**	.576**	.561**	.599**	.822**	N.A	N.A	N.A	N.A	N.A
*TAS – EOT*^*a*^	.077	.330**	.104	.104	.198**	N.A	N.A	N.A	N.A	N.A
*TAS – Alexithymia*^*a*^	.764**	.611**	.672**	.600**	.833**	N.A	N.A	N.A	N.A	N.A
*ERQ – ES*^*b*^	.660**	.506**	.433**	.537**	.687**	.646**	.385**	.204**	.359**	.486**
*ERQ – CR*^*b*^	–.113	–.278**	–.092	–.225**	–.216**	–.131*	–.119*	–.092	–.193**	–.168**

All correlations with ** are significant at a level of *p* < .001. Correlations with * are significant at a level of *p* < .05. KFS  (ger.: *Kölner Fragebogen zur Sprachlosigkeit*) Cologne Questionnaire on Speechlessness; *GED* general emotion description, *EA* emotional awareness; *EU* emotional uncertainty, *PED* positive emotion description, *TAS* toronto alexithymia scale; *DIF* difficulty in identifying emotions, *DDF* difficulty in describing emotions, *EOT* external-oriented thinking style, *ERQ* emotion regulation questionnaire, *ES* expressive suppression, *CR* cognitive reappraisal, *N.A.* not available; a = Sample 1: *N* = 203; b = Sample 1: *N* = 208/Sample 2: *N* = 381

## Discussion

Aim of this paper was to outline the item construction as well as the validation of the *Cologne Questionnaire on Speechlessness* (KFS; ger.: *Kölner Fragebogen zur Sprachlosigkeit*), as a new psychometric survey instrument to capture the construct of speechlessness in emotional distress and emotional dysregulation.

The KFS access individual’s non-speaking or silence in situations with an emotional context as well as potential dysregulation of one's own emotions, expressed in an avoidance of dealing with one's own emotions or difficulties in identifying them using a total of 19 items.

The EFAs conducted within each sample due to the exploratory nature of speechlessness identified a total of four different factorial structures, of which one four-factorial structure yielded the best results after confirmatory testing (cf. Table 2 and Fig. 2). The four-factorial structure of the KFS could be confirmed on the sample of oncologically diagnosed individuals with acceptable values in the model fit (CFI = 0.935; TLI = 0.915;RMSE = 0.057). Within the sample of the normal population, the criteria were minimally missed (CFI = 0.887; TLI = 0.853;RMSE = 0.077). In addition, individual factors of the four-factorial model showed high correlations among each other, but the test of a single-factorial model clearly failed to meet the quality criteria. A comparison of the model fit of the four-factorial structure of the KFS of the normal population sample with the goodness-of-fit criteria of the TAS-20 (Bagby et al., 1994) as a comparable survey instrument for clinical and nonclinical use to assess latent traits in the context of emotions also shows values below the required range (cf. Müller et al., 2003) of the literature (Hu & Bentler, 1995; Whittaker, 2016). Despite this, use of the TAS-20 (Bagby et al., 1994) proceeds in a wide range of settings (Bagby et al., 2020). In accordance with the common use of this survey instrument and its use in practice, the application of the KFS also can be recommended despite a lower model fit within the normal population.

The KFS subscales constructed using the factorial model can be placed in a theoretical context by correlating them to the subscales of the TAS-26 (Kupfer et al., 2001) and ERQ (Abler & Kessler, 2009). Considering the items of the GED subscale, there is a difficulty in expressing one’s own emotions over all items. This difficulty is clearly correlated with a suppression of one’s emotions and a potential somatic dysregulation of one’s emotions (alexithymia). Suppresion of emotion is correlated with physiological reactions (e.g. higher cortisol levels, increased respiratory sinus arrhythmia; Gross & Levenson, 1993, 1997; Butler et al., 2006) and an increased use of cognitive resources to suppress emotional expression reduced the speech production (Roche & Arnold, 2018). The presence of emotional dysregulation inhibits the perception and thus the description of one’s own emotions, which is why the person appears speechless (Goerlich-Dobre et al., 2014). It could be hypothesized, that individuals with a higher value on this scale might have problems in the general emotion description in words and therefore can be described as speechless. Considering the items of the sub-scale “Emotional Awareness” and the correlation to the sub-scales of the TAS-26 (Kupfer et al., 2001) and the ERQ (Abler & Kessler, 2009), potential conclusions can be drawn on the style of thinking and the associated perception of one’s own emotions and their linguistic expression. Based on the correlation to the EOT subscale of the TAS-26 (Kupfer et al., 2001), it can be hypothesized that an external way of thinking reduces perception because the person distances himself from his own emotions and therefore does not name them. Another assumption lies in the thinking style of individuals. Research indicates, that low levels of trait emotional intelligence are correlated to maladaptive coping styles (Ke & Barlas, 2020). It can be hypothesized that thinking styles, including emotional intelligence, are related to speechlessness. The subscales EU and PED show a similar connection to a dysfunctional description and perception of one's own emotions as well as an avoidance of expressing one's own emotions (ES). Unlike the GED, there is a nuanced difference within the item formulation of the scales. The EU subscale measures a person's confusion about their own emotions. It can be assumed that this confusion reduces verbal expression, as additional cognitive resources are required to reduce the "emotional confusion" that is a process associated with reduced verbal expression (cf. Johnson et al., 1996). The PED subscale records the description of one's own emotions in positive situations. Research indicates that positive emotions have an impact on language. Depending on the positive emotion experienced, this influences the speech rate (Kamiloğlu et al., 2020). Within these situations, the person's focus may be on the emotional stimuli, which can reduce the speech production and therefore leads to reduced speech or speechlessness about one’s emotions. However, it cannot be excluded that the correlations are due to a close semantic alignment of the items with the TAS-26 (Kupfer et al., 2001) and the PAQ (Preece et al., 2018). A positive or negative correlation between the scales of the ERQ (Abler & Kessler, 2009) and measurement procedures of the Toronto model of alexithymia (Taylor et al., 1997) has already been established several times (cf. Schiewer et al., 2022). In this case, the correlation would only confirm already recorded relationships between the concepts of expressive suppression and alexithymia. At the same time, a necessity arises from this, the construction of an independent, superordinate factor, which can be defined as (emotional) speechlessness.

Another important aspect, which was assessed in the context of the validation of the KFS, is the existing measurement invariances between the samples. The analyses performed explicitly for the female part indicate a difference between persons of the normal population and individuals with oncological diseases. Measurement invariances at all four levels (cf. Putnick & Bornstein, 2016) indicate differences across survey settings. One reason for this could be the generally higher psychological burden of oncological patients (Mehnert et al., 2018). Additionally, cancer patients show an increased prevalence of alexithymia (DeVries et al., 2012). These factors may explain both the recorded measurement invariances between groups and the elevated subscale scores of the clinical setting sample.

In conclusion, based on the results presented, the use of the *Cologne Questionnaire on Speechlessness* (KFS) can be recommended, taking into account potential limitations of the survey instrument. The phenomenon of speechlessness can be mapped in the dimensions of the KFS as well as their clear relationship to the TAS-26 and ERQ scales. The results of the CFA show resilient factors, on the basis of which a classification of difficulties and processes of emotional perception and evaluation is possible. Overarching, the factors can be used in the further research process as a basis for the development of an independent and more comprehensive construct of speechlessness, also outside the emotional context.

### Limitation and outlook

The validation of the questionnaire is based on some limitations. All samples were collected in an online survey. This survey format makes it possible to generate a high number of cases with little effort. At the same time, this form of survey, which belongs to the convenience sampling methods, is susceptible to potential bias. Despite technical restrictions, double participation of individuals cannot be excluded. Likewise, further selection effects and biases due to a lack of randomization of the samples cannot be excluded. Due to the ongoing burden of the SARS-CoV-2 pandemic during the survey, biases in the data cannot be ruled out here either. Surveys within a sample of the normal population, embodied in the form of the employees and students of the University Hospital Cologne and the University of Cologne, as well as a sample of persons with oncological diseases, attempted to include both clinical and non-clinical samples in the validation process.

In addition, the results of testing for measurement invariances between female participants with and without cancer show significant differences in model fit, which indexes differences between survey settings. Here, it is necessary in upcoming studies to decisively discuss differences between clinical and non-clinical samples.

Conclusions drawn from the results should always be made taking into account the high proportion of female respondents in both samples. Although the strong correlation of the (sub)scales of the KFS to the TAS-26 (Kupfer et al., 2001) and the ERQ (Abler & Kessler, 2009) confirm the assumption of the working group around Schiewer et al. (2022), these should be set complementary to other related concepts of psychology (depression, anxiety, etc.) and examined on a larger sample of the normal population. Furthermore, it should be examined whether the concept of speechlessness is a situational factor or a latent, persistent personality trait.

In addition, a comprehensive review of different theoretical concepts and constructs is needed to capture speechlessness as a whole in individual components as well as the dimensions of intentional and non-intentional speechlessness (Berger, 2004). With regard to this process, the KFS should be seen as a first survey instrument and starting point for future research in the field of speechlessness.

## Supplementary Information

Below is the link to the electronic supplementary material.

Supplementary file1 (DOCX 27 KB)
