# Supplementary material for: Cologne questionnaire on speechlessness: Development and validation
Source: Curr Psychol. 2022 Dec 11:1–12. Online ahead of print. doi: 10.1007/s12144-022-04102-x (PMC9741759; doi:10.1007/s12144-022-04102-x)
Supplement: Supplementary file 1 — Supplementary file1 (DOCX 27 KB) [file 12144_2022_4102_MOESM1_ESM.docx]

**Electronic Supplementary Material (ESM)**

**ESM Table E1.** Sociodemographic characteristics of the validation sample (sample 2; n = 520)

|  | | ***Sample 1***  ***(N = 307)*** | | | ***Sample 2***  ***(N = 621)*** | | | ***Sample 3***  ***(N = 365)*** | | | ***Combined Sample***  ***(N = 1293)*** | | |
| --- | --- | --- | --- | --- | --- | --- | --- | --- | --- | --- | --- | --- | --- |
|  |  | **N**  **(M)** | **%**  **(SD)** | **RG** | **N**  **(M)** | **%**  **(SD)** | **RG** | **N**  **(M)** | **%**  **(SD)** | **RG** | **N**  **(M)** | **%**  **(SD)** | **RG** |
| Age | *Age in years* | (36.78) | (12.7) | 18 – 68 | (55.8) | (11.57) | 20 – 84 | (34.47) | (13.67) | 18 – 65 | (45.28) | (16.17) | 18 – 84 |
|  | *missing* | [105] | [34.2%] |  | [101] | [16.3%] |  | [18] | [4.9%] |  | [224] | [17.3%] |  |
| Gender | *male* | 33 | 16.3% |  | 114 | 21.9% |  | 60 | 17.3% |  | 207 | 19.4% |  |
|  | *female* | 169 | 83.7% |  | 406 | 78.1% |  | 286 | 78.4% |  | 861 | 80.6% |  |
|  | *missing* | [105] | [34.2%] |  | [101] | [16.3%] |  | [19] | [5.2%] |  | [225] | [17.4%] |  |
| Marital status | *single* | 40 | 19.8% |  | 56 | 10.8% |  | 120 | 34.6% |  | 216 | 20.2% |  |
|  | *married* | 73 | 23.8% |  | 331 | 63.7% |  | 79 | 22.8% |  | 483 | 45.2% |  |
|  | *divorced* | 9 | 4.5% |  | 60 | 11.5% |  | 17 | 4.9% |  | 86 | 8% |  |
|  | *widowed* | - | - |  | 13 | 2.5% |  | 1 | .3% |  | 14 | 1.3% |  |
|  | *in permanent partnership* | 77 | 38.1% |  | 55 | 10.6% |  | 129 | 37.2% |  | 261 | 24.4% |  |
|  | *registred partnership* | 2 | 1% |  | 3 | .6% |  | 1 | .3% |  | 6 | .6% |  |
|  | *registered partnership dissolved* | - | - |  | 1 | .2% |  | - | - |  | 1 | .1% |  |
|  | *partner deceased* | 1 | .5% |  | 1 | .2% |  | - | - |  | 2 | .2% |  |
|  | *missing* | [202] | [34.2%] |  | [101] | [16.3%] |  | [18] | [4.9%] |  | [224] | [17.3%] |  |
| Educational level | *Secondary school diploma* | 26 | 12.9% |  | 211 | 40.6% |  | 58 | 13.8% |  | 285 | 26.6% |  |
|  | *High school diploma* | 37 | 18.3% |  | 59 | 11.3% |  | 125 | 35.9% |  | 221 | 20.7% |  |
|  | *Vocational training* | 27 | 13.4% |  | 96 | 18.5% |  | 57 | 16.4% |  | 180 | 16.8% |  |
|  | *Bachelor’s degree* | 27 | 13.4% |  | 21 | 4% |  | 44 | 12.6% |  | 92 | 8.6% |  |
|  | *Master’s degree* | 36 | 17.8% |  | 35 | 6.7% |  | 34 | 9.8% |  | 105 | 9.8% |  |
|  | *Diploma* | 17 | 8.4% |  | 83 | 16% |  | 16 | 4.6% |  | 116 | 10.8% |  |
|  | *Promotion/PhD* | 32 | 13.4% |  | 15 | 2.9% |  | 24 | 6.9% |  | 71 | 6.6% |  |
|  | *missing* | [105] | [35.2%] |  | [101] | [16.3%] |  | [17] | [4.7%] |  | [223] | [17.2%] |  |
| Cancer diagnosis period | *≤ 12 months* | - | - |  | 146 | 27.8% |  | - | - |  | 146 | 11.3% |  |
|  | *12 > months and ≤ 5 years* | - | - |  | 231 | 44% |  | - | - |  | 231 | 17.9% |  |
|  | *> 5 years* | - | - |  | 148 | 28.2% |  | - | - |  | 148 | 11.4% |  |
|  | *missing* | - | - |  | [96] | [15.5%] |  | - | - |  | [96] | [7.4%] |  |

***Note.*** RG = Range; [] = Values refer to the number of all participants in the sample.

**ESM Table E2.** Selected Items

| **No.** | **Item difficulty** | **Discriminant power** | **Cronbach’s Alpha** |
| --- | --- | --- | --- |
| 1 | .24 | .59 | .923 |
| 2 | .26 | .68 |  |
| 3 | .34 | .65 |  |
| 4 | .16 | .61 |  |
| 5 | .34 | .59 |  |
| 6 | .32 | .37 |  |
| 7 | .39 | .62 |  |
| 8 | .20 | .63 |  |
| 9 | .34 | .67 |  |
| 10 | .21 | .61 |  |
| 11 | .34 | .75 |  |
| 12 | .28 | .60 |  |
| 13 | .32 | .78 |  |
| 14 R | .32 | .56 |  |
| 15 R | .35 | .55 |  |
| 16 R | .31 | .49 |  |
| 17 R | .43 | .47 |  |
| 18 R | .45 | .46 |  |
| 19 | .24 | .71 |  |

**Note.** Sample 1; N = 226; R = recoded inverse

**ESM Table E3.** Extratected pattern matrix of the EFA.

|  | **Sample 1 (N = 226)** | | | | **Sample 2 (N = 469)** | | | **Sample 3 (N = 232)** | | | **Combined sample (N = 927)** | | |
| --- | --- | --- | --- | --- | --- | --- | --- | --- | --- | --- | --- | --- | --- |
|  | **F1** | **F2** | **F3** | **F4** | **F1** | **F2** | **F3** | **F1** | **F2** | **F3** | **F1** | **F2** | **F3** |
| *KFS I1* |  |  | **.704** |  | **.643** |  |  | **.831** |  |  |  |  | **.676** |
| *KFS I2* | .336 |  | **.538** |  | **.652** |  |  | **.706** |  |  | .405 |  | **.478** |
| *KFS I3* | **.716** |  | .360 |  | **.740** |  |  | **.680** |  |  | **.480** |  | .478 |
| *KFS I4* |  |  | .353 | **.729** | **.737** |  |  | **.430** |  |  | **.550** |  |  |
| *KFS I5* | **.418** |  | .374 |  | **.677** |  |  | **.589** |  |  | **.467** |  | .413 |
| *KFS I6* |  |  | **.855** |  | .364 |  | **.659** | **.790** |  |  |  |  | **.802** |
| *KFS I7* | **.785** |  |  |  | **.821** |  |  | .341 |  | **-.599** | **.735** |  |  |
| *KFS I8* |  |  |  | **.796** | **.768** |  |  |  |  | **-.631** | **.734** |  |  |
| *KFS I9* | **.718** |  |  |  | **.808** |  |  | .395 |  | **-.453** | **.685** |  |  |
| *KFS I10* |  |  |  | **.830** | **.800** |  |  |  |  | **-.527** | **.740** |  |  |
| *KFS I11* | **.736** |  |  |  | **.760** |  |  |  |  | **-.890** | **.810** |  |  |
| *KFS I12* | **.714** |  |  |  | **.607** |  | -.436 |  |  | **-.743** | **.781** |  |  |
| *KFS I13* | **.731** |  |  |  | **.800** |  |  | .313 |  | **-.651** | **.766** |  |  |
| *KFS I14* |  | **.836** |  |  |  | **.795** |  |  | **.844** |  |  | **.818** |  |
| *KFS I15* |  | **.777** |  |  |  | **.742** |  |  | **.806** |  |  | **.773** |  |
| *KFS I16* |  | **.809** |  |  |  | **.766** |  |  | **.780** |  |  | **.776** |  |
| *KFS I17* |  | **.832** |  |  |  | **.800** |  |  | **.852** |  |  | **.820** |  |
| *KFS I18* |  | **.800** |  |  |  | **.779** |  |  | **.862** |  |  | **.802** |  |
| *KFS I19* | .315 | **.405** |  |  | **.481** | .343 |  |  | **.530** |  | .380 | **.413** |  |

**Notes.** Values < .3 are not shown for better readability. Exploratory factor analysis using the principal components method based on an eigenvalue > 1 and an oblique rotation (oblimin, direct) with delta = 0.

**ESM Table E4.** Scale reliability of four-factorial-structure of the KFS (model 1)

|  | Sample 1  (N = 307) | | Sample 2  (N = 621) | | Sample 3  (N = 365) | | Combined Sample  (N = 1293) | |
| --- | --- | --- | --- | --- | --- | --- | --- | --- |
|  | N | Cronbach’s Alpha | N | Cronbach’s Alpha | N | Cronbach’s Alpha | N | Cronbach’s Alpha |
| GED | 226 | .893 | 469 | .890 | 232 | .873 | 927 | .890 |
| EA |  | .874 |  | .822 |  | .881 |  | .853 |
| EU |  | .771 |  | .703 |  | .768 |  | .750 |
| PED |  | .841 |  | .839 |  | .846 |  | .850 |
| Total score |  | .923 |  | .902 |  | .919 |  | .916 |

**Note.** For the internal consistency calculation, only those cases were used that provided complete information on the KFS and allowed a calculation of the total score.

**ESM Table E5.** Indifferences between male and female indiviuals in the four-factorial-structure of the KFS (model 1)

|  | **male**  **(N = 207)** | **female**  **(N = 861)** |
| --- | --- | --- |
| *χ²* | 408.445 | 789.566 |
| *χ²/df - ratio* | 2.798 | 5.408 |
| *CFI* | .883 | .914 |
| *TLI* | .848 | .889 |
| *RMSEA* | .066*** | .072 |

**Note.** Female and male individuals were not separated in cancer and non-cancer. * p < .05; ** p < .01; *** p < .001

**ESM Table E6.** Partial scalar invariance.

| Factor/ Subscale | Item | χ² | df | χ²_Scalar_ - χ²_tested intercept_ | df_Scalar_ – df_tested intercpet_ | p-value (sig.) |
| --- | --- | --- | --- | --- | --- | --- |
| GED | 3 | 1606.2 | 322 | .8 | 1 | .371 |
|  | 5 | 1594.2 | 322 | 12.8 | 1 | < .001 |
|  | 7 | 1606 | 322 | 1 | 1 | .317 |
|  | 9 | 1607 | 322 | 0 | 1 | 1,000 |
|  | 11 | 1606.9 | 322 | .1 | 1 | .752 |
|  | 12 | 1603.6 | 322 | 3.4 | 1 | .065 |
|  | 13 | 1607 | 322 | 0 | 1 | 1.000 |
| EA | 14R | 1607 | 322 | 0 | 1 | 1.000 |
|  | 15R | 1605.5 | 322 | 1.5 | 1 | .221 |
|  | 16R | 1605.9 | 322 | 1.1 | 1 | .294 |
|  | 17R | 1605.2 | 322 | 1.8 | 1 | .80 |
|  | 18R | 1599 | 322 | 8 | 1 | .005 |
|  | 19 | 1571.4 | 322 | 35.6 | 1 | < .001 |
| EU | 1 | 1606 | 322 | 1 | 1 | .317 |
|  | 2 | 1592.6 | 322 | 14.4 | 1 | < .001 |
|  | 6 | 1554.2 | 322 | 52.8 | 1 | < .001 |
| PED | 4 | 1556.9 | 322 | 50.1 | 1 | < .001 |
|  | 8 | 1601.6 | 322 | 5.4 | 1 | .020 |
|  | 10 | 1599.9 | 322 | 7.1 | 1 | .008 |

**Note.** Reference group N = 406 (cancer indiviuals); χ²(323)_Scalar_ = 1607

**ESM Table E7.** Measurement invariences for female participants.

|  | **χ²** | **df** | **CFI** | **Δχ²** | **Δdf** | **p (sig.)** | **ΔCFI** | **Calculations** |
| --- | --- | --- | --- | --- | --- | --- | --- | --- |
| *Baseline* | 1008.7 | 292 | .903 | - | - | - | - | - |
| *Metric* | 1433.7 | 308 | .847 | 425 | 16 | < .001 | .056 | Metric – Baseline |
| *Scalar* | 1607 | 323 | .826 | 173.3 | 15 | < .001 | .021 | Scalar – Metric |
| *Partial invariance^a^* | 1452.2 | 317 | .846 | 154.8 | 6 | < .001 | .02 | Partial invariance – Scalar |
| *Partial invariance intercepts* | 1616.5 | 337 | .826 | 164.3 | 20 | < .001 | .02 | Partial invariance intercepts – Partial invariance intercepts |

**Note.** Reference group N = 406 (cancer indiviuals); a = partial invariance model intercepts for Item 2, 4, 5, 6 and 19 were freely estimated across groups
